# Fertility preservation and assisted reproductive strategies in endometrial cancer patients with lynch syndrome

**DOI:** 10.3389/fonc.2025.1630301

**Published:** 2025-07-21

**Authors:** Junhan Liu, Ying Zheng, Jianhong Liu

**Affiliations:** ^1^ Department of Obstetrics and Gynecology, West China Second Hospital of Sichuan University, Chengdu, Sichuan, China; ^2^ Key Laboratory of Obstetrics and Gynecologic and Pediatric Diseases and Birth Defects of Ministry of Education, West China Second Hospital of Sichuan University, Chengdu, Sichuan, China

**Keywords:** lynch syndrome, fertility-sparing treatment, endometrial carcinoma, assisted reproductive technology (ART), preimplantation testing for single gene genetic disorders (PGT-M)

## Abstract

Patients with LS-EC can be treated with progestin-based fertility-sparing treatment under close monitoring, and pregnancy is recommended as soon as possible after complete remission (CR) of the disease, with assisted reproduction, supplemented by PGT-M, to minimize the probability of inheritance of the disease in the offspring. Radical surgery for endometrial cancer is recommended as soon as possible after completion of childbearing to minimize recurrence. The role of assisted reproductive technologies (ART) and preimplantation genetic testing for monogenic disorders (PGT-M) was explored. For patients achieving CR, early initiation of ART, especially IVF with frozen-thawed embryo transfer (FET), was associated with improved reproductive outcomes. PGT-M proved valuable in preventing the transmission of pathogenic MMR variants to offspring. Early use of ART and integration of PGT-M are critical for maximizing reproductive success while minimizing oncologic and hereditary risks.

## Introduction

The incidence of endometrial cancer has been rising in recent years, and a small proportion of cases are associated with Lynch syndrome–related endometrioid adenocarcinoma. Lynch syndrome, also known as hereditary non-polyposis colorectal cancer (HNPCC), is an autosomal dominant disorder characterized by germline mutations in mismatch repair (MMR) genes such as *MLH1*, *MSH2*, *MSH6*, and *PMS2*. Offspring of affected individuals have a 50% risk of inheriting the same pathogenic mutation. Lynch syndrome increases the risk of various malignancies, with endometrial cancer (EC) being the most common extraintestinal tumor in women. Notably, EC is the first diagnosed tumor in 40% to 60% of women with Lynch syndrome, making it a potential sentinel cancer in this population. Compared with sporadic EC, patients with Lynch syndrome–associated EC tend to be younger, have lower BMI, typically lack estrogen-related symptoms and signs, and often present with tumors located in the lower uterine segment.

There is currently no consensus on whether fertility-preserving treatment is appropriate for patients with LS-associated EC. Following complete remission, it is recommended that patients pursue pregnancy as soon as possible, with assisted reproductive technology (ART) being the preferred option. ART can facilitate pregnancy while maintaining treatment, shorten the time to conception, and reduce the risk of recurrence. However, for patients with Lynch syndrome, spontaneous pregnancy carries a high risk of hereditary disease transmission. Thus, the use of ART in fertility-preserving management for this population remains controversial and warrants further investigation. This article explores the safety, feasibility, and practical implementation of ART in the context of Lynch syndrome, based on the latest international research and expert opinions.

## Lynch syndrome and endometrial carcinoma

Lynch syndrome is primarily caused by germline mutations in DNA mismatch repair (MMR) genes, most commonly involving *MLH1*, *MSH2*, *MSH6*, and *PMS2* (1). These mutations result in defective MMR function, leading to microsatellite instability (MSI) and promoting genome-wide mutation accumulation—hallmark molecular features of Lynch syndrome–associated tumors (2). In women with Lynch syndrome, EC is the second most common malignancy after colorectal cancer, with a particularly high incidence among MSH6 and MSH2 mutation carriers. The lifetime risk of EC in this population is estimated at 40–60%, markedly exceeding the 3% risk observed in the general population (3). Moreover, Lynch syndrome–associated endometrial carcinoma (LS-EC) differs markedly from sporadic cases in terms of epidemiology, pathological characteristics, anatomical distribution, and prognosis. It typically presents at a younger age, with a mean onset of 46–49 years, and approximately 60% of cases occur before the age of 50. Histologically, most tumors are type I endometrioid adenocarcinomas; however, they often lack estrogen dependence and are more likely to exhibit deep myometrial invasion and lymphovascular space involvement. LS-EC also demonstrates a predilection for the lower uterine segment, with up to 29% of endometrioid carcinomas in this region linked to Lynch syndrome. Although the overall 5-year survival rate may reach 88%, the prognosis significantly worsens in the event of recurrence or metastasis (4).

## Fertility-sparing treatment and follow-up

According to the latest NCCN guidelines (5), fertility-sparing treatment is recommended only for carefully selected patients who meet strict inclusion criteria: well-differentiated (grade 1) endometrioid endometrial carcinoma (EEC); tumor confined to the endometrium as confirmed by imaging; absence of extrauterine or metastatic disease; age under 40 years with a strong desire for future fertility; no contraindications to hormonal therapy or pregnancy; and full informed consent. The 2023 guidelines jointly issued by ESGO, ESHRE, and ESGE (6) acknowledge that patients with Lynch syndrome may have a concurrent hyperestrogenic state contributing to EC development and may be considered for progestin-based therapy. Although the carcinogenic mechanisms in Lynch syndrome–associated endometrial cancer (LS-EC) differ from those of sporadic cases, progestin therapy may still be effective in select patients. High-dose progestins remain the first-line agents for conservative management, owing to their potential to induce regression of endometrial lesions even in the context of mismatch repair deficiency. However, the presence of mismatch repair deficiency (MMR-D) is associated with a higher risk of treatment resistance and recurrence, and surgical lesion excision via hysteroscopy is emphasized as a critical component of successful management. Recent reports also suggest that while complete remission can still be achieved in select LS patients, the recurrence rate following progestin therapy ranges widely—from 20.1% to 100%—reflecting significant variability and clinical uncertainty (7, 8). To address these limitations, several alternative or adjunctive strategies have been explored. The levonorgestrel-releasing intrauterine system (LNG-IUS) offers a locally targeted progestin delivery method with reduced systemic side effects and has shown favorable outcomes in patients with complex atypical hyperplasia and early-stage endometrial carcinoma (6, 9, 10). Combination therapies, such as gonadotropin-releasing hormone agonists (GnRH-a) with aromatase inhibitors like letrozole, have demonstrated potential in suppressing estrogen production and tumor progression, particularly in obese or progestin-resistant individuals (11). Importantly, given the high prevalence of microsatellite instability-high (MSI-H) and MMRd status in LS-EC, immune checkpoint inhibitors have emerged as a promising therapeutic modality. Clinical trials have shown that agents such as pembrolizumab and dostarlimab yield objective response rates ranging from 34% to 42% in patients with advanced or recurrent dMMR endometrial cancer (12). While their role in fertility-sparing treatment is still investigational, immunotherapy may serve as a bridge to definitive therapy or as a component of individualized management plans. To date, fertility-preserving treatments in LS-EC have primarily involved single-gene mutations(e.g., isolated MSH6 or MLH1 variants), and little is known about the efficacy of such approaches in patients with concurrent pathogenic mutations involving other oncogenic pathways. Given these limitations, international guidelines such as ESGO/ESHRE/ESGE recommend that fertility-sparing therapy in patients with LS be considered only after thorough multidisciplinary evaluation, with individualized risk-benefit assessment and rigorous surveillance throughout treatment and follow-up. To conclude, LS-EC is not deemed an absolute contraindication to fertility preservation.

According to ESGO/ESHRE/ESGE guideline, fertility-preserving treatment for EC should be conducted in three-month phases starting from the initiation of medication. After each phase, the treatment’s effectiveness should be evaluated through imaging techniques to assess the endometrium and pelvic region, with endometrial tissue samples collected for pathological analysis, predominantly via hysteroscopy.

Consolidation and maintenance therapy are particularly important components of fertility-sparing management in patients with endometrial cancer, especially those with Lynch syndrome (13). For patients who achieve CR but do not intend to conceive in the near term, maintenance therapy with a continuous low-dose oral progestin or a levonorgestrel-releasing intrauterine system (LNG-IUS) may be recommended. This method ensures continuous local progestin delivery with high treatment compliance and has been associated with a reduced risk of recurrence. The LNG-IUS thus offers a practical and effective option for long-term disease control during the interval between remission and planned pregnancy.

## Assisted reproductive strategies

Patients with LS-EC face not only oncologic uncertainty regarding the safety and efficacy of conservative treatment but also complex reproductive considerations, as each offspring has a 50% risk of inheriting a pathogenic variant (14). In such cases, preimplantation or prenatal genetic testing may be considered to mitigate the risk of transmission.

For patients with LS-EC undergoing fertility-preserving treatment, early initiation of assisted reproductive therapy following CR is recommended. Evidence suggests that attempting conception within six months post-CR improves pregnancy outcomes, whereas treatment delays may increase the risk of recurrence (15). Among available reproductive strategies, *in vitro* fertilization (IVF) combined with frozen-thawed embryo transfer (FET) is considered optimal, as IVF improves conception rates and FET minimizes exposure to supraphysiologic hormone levels, thereby potentially reducing recurrence risk (16, 17).

For mutation carriers, preimplantation genetic testing for monogenic disorders (PGT-M) is advised to prevent transmission of pathogenic variants associated with Lynch syndrome. PGT-M enables the identification and selection of unaffected embryos through genetic analysis during IVF, thereby reducing the likelihood of transmitting the disorder to offspring (18). Current best practice involves combining direct mutation detection with linkage analysis using single nucleotide polymorphisms (SNPs) or short tandem repeats (STRs) to establish informative haplotypes in families with adequate genetic data, thereby enhancing diagnostic accuracy and mitigating allele dropout (ADO) risk (19).

Advancements in third-generation sequencing technologies have further enabled direct haplotype construction through long-read single-molecule sequencing, offering a reliable solution for patients with *de novo* mutations or incomplete family data. However, as PGT-M cannot ensure absolute accuracy due to the potential for embryonic mosaicism or sampling error, confirmatory prenatal diagnostic testing remains essential following implantation.

## Discussion

Current evidence on LS-EC remains limited, with most studies restricted to case reports and a lack of high-quality, large-scale data to inform clinical decision-making (20). Although LS-EC differs from sporadic EC in its molecular pathogenesis, it is not considered an absolute contraindication to fertility preservation. Some patients may exhibit a hyperestrogenic state, allowing for regression of endometrial lesions with progestin therapy; thus, fertility-sparing treatment may be considered following comprehensive counseling and informed consent (21). Given the complexity of care in this setting, a multidisciplinary team (MDT) approach is essential. The MDT should include experts in gynecologic oncology, reproductive endocrinology, assisted reproduction, radiology, pathology, and medical genetics to collaboratively assess each case and formulate individualized management plans based on consensus and clinical expertise.

According to the 2023 FIGO guidelines (22), comprehensive molecular classification is recommended for all EC patients. The appropriateness of fertility-sparing therapy in patients with MMR-D remains controversial; such cases require cautious selection, rigorous monitoring, and shared decision-making. As LS carries a 50% risk of transmission to offspring, PGT-M is recommended during assisted reproduction to prevent vertical transmission of pathogenic variants (23). In patients with *de novo* mutations or incomplete pedigree data, conventional linkage-based haplotype construction may be infeasible (24). In such cases, third-generation sequencing technologies offer a robust alternative for direct haplotyping and PGT-M (25).

Currently, universal genetic screening for Lynch syndrome in asymptomatic women with primary infertility is not recommended (26). From a health economics standpoint, such an approach is not considered cost-effective in the general infertile population due to the relatively low prevalence of Lynch syndrome and the high cost associated with comprehensive germline testing. Instead, a risk-stratified, clinically driven strategy is more appropriate. In patients with unexplained infertility, a thorough evaluation should first be conducted to rule out common etiologies, including endocrine, tubal, ovulatory, and uterine factors. In cases where abnormal uterine bleeding persists or imaging reveals suspicious endometrial findings, hysteroscopic endometrial sampling may be indicated to obtain histological evidence. If endometrial pathology demonstrates atypical hyperplasia or malignancy, or if there is a significant personal or family history of early-onset cancers (such as colorectal, endometrial, or ovarian cancer diagnosed before age 50), targeted germline testing for mismatch repair (MMR) gene mutations, including Lynch syndrome-associated genes, should be considered. This selective approach facilitates early identification of high-risk individuals while minimizing unnecessary genetic testing in low-risk populations, thereby optimizing both clinical outcomes and healthcare resource utilization. Additionally, In line with the 2024 NCCN guidelines (27), gynecologic evaluation should be considered in women diagnosed with colorectal cancer, particularly those under 50 years of age or with a family history suggestive of Lynch syndrome. For individuals carrying pathogenic variants in MMR genes, periodic endometrial sampling every 1–2 years starting at age 30–35 is recommended. In selected high-risk cases, prophylactic hysterectomy with bilateral salpingo-oophorectomy may also be considered. These strategies enable early detection of endometrial pathology and are essential components of comprehensive risk management in Lynch syndrome carriers.

Pregnancy and live birth rates among patients with LS-EC remain relatively low (8, 28, 29). Thus, fertility assessment should be conducted prior to initiating conservative treatment, and meticulous endometrial preservation during hysteroscopic procedures is critical. Once CR is achieved, pregnancy should be pursued promptly to optimize outcomes while minimizing the window for recurrence. Marton (30) et al. reported two cases of LS-associated endometrial cancer (LS-EC) in which patients successfully conceived and delivered following standardized fertility-sparing treatment. While these outcomes are encouraging, the evidence remains limited due to the small sample size and lack of long-term oncologic follow-up. As such, these preliminary findings should be interpreted with caution and cannot yet be extrapolated to broader clinical practice. Larger, multicenter prospective studies and registry-based data are warranted to establish the safety, efficacy, and generalizability of fertility-preserving strategies in this unique patient population. Standardized follow-up with both oncologic surveillance and reproductive support is essential to reduce relapse risk. Additionally, long-term monitoring should include screening for LS-associated metachronous malignancies.

For patients with EC who achieve CR following fertility-sparing treatment, current guidelines recommend initiating ART as early as possible to maximize the chances of pregnancy and minimize the risk of recurrence. In LS-EC patients with germline mutations, ART—particularly when combined with PGT-M—is considered the preferred approach to simultaneously reduce the risk of disease transmission and recurrence (31). PGT-M offers several advantages in the context of Lynch syndrome (26, 32, 33): (1) high specificity, enabling accurate detection of known pathogenic variants such as *MSH6 c.3261dupC* for targeted embryo selection (34); (2) effective genetic prevention by selecting embryos free of the mutation, thereby halting vertical transmission; (3) psychological benefits for future offspring by eliminating the need for lifelong cancer surveillance and associated anxiety; and (4) ethical advantages by ensuring reproductive safety without relying on prenatal diagnosis followed by medical termination. PGT-M has demonstrated efficacy in reducing transmission rates in families with hereditary cancer syndromes and represents a valuable tool in reproductive planning for LS-EC patients.

PGT-M offers a preventive strategy to avoid transmission of pathogenic MMR gene variants in LS-EC patients pursuing assisted reproductive technologies. Meanwhile, several limitations warrant careful consideration in clinical decision-making. The success of PGT-M depends on multiple factors, including maternal age, ovarian reserve, and embryo quality—parameters that may be adversely affected by prior hormonal therapy or delays in fertility planning. Moreover, cumulative live birth rates after PGT-M are often lower than those of conventional IVF, particularly in women of advanced age or with comorbidities (35). High financial costs and limited insurance coverage further restrict access, with many patients requiring multiple IVF cycles to obtain transferrable, mutation-free embryos, especially in autosomal dominant conditions like Lynch syndrome. The accuracy of PGT-M depends on robust linkage analysis and high-quality DNA amplification, which may be complicated in cases with low embryo yield or poor-quality biopsies. Mosaicism and allele drop-out can also affect diagnostic reliability, potentially leading to false-negative or false-positive results (36). In light of these considerations, PGT-M should be offered as part of comprehensive reproductive counseling by a multidisciplinary team that includes reproductive endocrinologists, genetic counselors, and gynecologic oncologists. Transparent communication about the realistic success rates, financial implications, and ethical boundaries of PGT-M is critical in supporting informed decision-making for LS-EC patients and their families.

## Conclusion

Patients with LS-EC can be treated under close monitoring, and pregnancy is recommended as soon as possible after complete remission of the disease, with assisted reproduction, supplemented by PGT-M.
